# Reliability of diffusion weighted MR imaging in differentiating degenerative and infectious end plate changes

**DOI:** 10.2478/v10019-010-0006-z

**Published:** 2010-05-24

**Authors:** Ozgur Oztekin, Cem Calli, Omer Kitis, Zehra Hilal Adibelli, Cemal Suat Eren, Melda Apaydin, Mehmet Zileli, Taskin Yurtseven

**Affiliations:** 1 Radiology Department, Izmir Education and Research Hospital, Izmir, Turkey; 2 Department of Radiology, Ege University Medical School, Izmir, Turkey; 3 Radiology Department, Tepecik Education and Research Hospital, Izmir, Turkey; 4 Radiology Department, Ataturk Education and Research Hospital, Izmir, Turkey; 5 Department of Neurosurgery, Ege University Medical School, Izmir, Turkey

**Keywords:** Modic type 1 change, spondylodiscitis, magnetic resonance imaging, diffusion-weighted imaging, vertebral end-plate

## Abstract

**Background:**

The aim of the study was to investigate the value of diffusion weighted MR imaging in the diagnosis of Modic type 1 change, which may be confused with the acute infectious spondylodiscitis on conventional MR imaging.

**Patients and methods:**

Twenty-seven patients with erosive intervertebral osteochondrosis, Modic type 1 and 18 patients with spondylodiscitis were included in this retrospective study. All images were acquired using on 1.5 Tesla MR units. Lumbar spinal MR imaging of 45 patients were retrieved from a digital database of a radiology record system and evaluated by one experienced radiologist. Patients with Modic type 1 change had CT slices obtained from the diseased disc space and the affected vertebrae.

**Results:**

Bone marrow adjacent to the vertebral end plate in both Modic type 1 change and acute spondylodiscitis were hypointense on T1-weighted images. On T2-weighted images corresponding levels of vertebral end-plates showed hyperintense signal intensity in both group. All the patients with spondylodiscitis and Modic type 1 change were hyperintense and hypointense on diffusion-weighted MR images, respectively.

**Conclusions:**

Our findings suggest that diffusion weighted MR imaging is an useful method in differentiating Modic type 1 changes from acute spondylodiscitis, both of which may mimic each other, either on clinical or conventional MRI findings.

## Introduction

Vertebral end-plate abnormalities of the lumbar spine are commonly seen on MR images.^1^ Of these abnormalities most of them are frequently associated with degeneration.

Degenerative vertebral end-plate changes were first described independently by Roos *et al.*^2^ and Modic *et al.*^3^ as being a feature associated with the degenerative disk disease. These changes, also called as erosive intervertebral osteochondrosis (EIVO), were classified into three groups by Modic *et al.*^3^ Type 1 change is vascular granulation tissue, demonstrated as low signal intensity on T1-weighted images (T1WI) and high signal intensity on T2-weighted images (T2WI). They are associated with fissuring of cartilaginous end-plate and increased vascularity within the subchondral bone marrow on the histological examination.^4^ Type 2 change is fatty infiltration of the end-plates, demonstrated as hyper intense signal intensity on T1WI and hyper intense or isointense signal intensity on T2WI. In such cases biopsy reveals the fatty replacement of the marrow, which is thought to be the result of marrow ischemia.^4^ Type III changes consist of reduced signal intensity on both T1- and T2-weighted images representing bone sclerosis.^3^

Intervertebral disk space infections typically give rise to vertebral marrow oedema, manifesting as areas of low signal intensity on T1WI and high signal intensity on T2WI.^5^

Thereby, type 1 Modic changes may cause a diagnostic dilemma in patients with low back pain since sometimes it resembles the MRI features of spondylodiscitis.^6^

The diffusion weighted imaging (DWI) has recently been used in the spine by many authors, mainly for the differentiation of benign and malign oedema of the vertebral body.^7^–^9^ DWH might be also useful in the differential diagnosis of benign from malignant lesions in other organs.^10^ It has been reported that benign fracture oedema depicts hypoisointensity on DWI whereas malign infiltration of the vertebrae discloses hyperintensity. To distinguish the benign from the malignant differences is crucial to choose the right treatment.^11^ DWI has also been used in spondylodiscitis. It has been shown that DWI reveals hyperintensity in the affected vertebrae and the paravertebral infectious soft tissue in acute spondylodiscitis.^12^ The purpose of this investigation was to evaluate the usefulness of diffusion weighted MR imaging for the differentiation of Modic type 1 changes from acute spondylodiscitis, both of which may mimick each other, either on the clinical evaluation or conventional MRI findings.

## Patients and methods

### Patients

Forty-five patients (18 patients with acute spondylodiscitis and 27 patients with Modic type 1 change) who underwent lumbar MRI examinations between January 2001 and December 2008 were included after a review of a digital database of a radiology record system. They were identified from a total of 1400 MR imaging examinations of the lumbar spine with a low back pain performed during this time at our institution. Patients with signal abnormalities limited to having previous surgery, recent vertebral fracture, metastatic disease, and pregnancy were excluded from the study.

Spondylodiscitis were proven with CT-guided biopsy in 12 patients. In 6 patients the diagnosis of spondylodiscitis were based on laboratory findings.

The diagnosis of Modic type 1 change were proven either by clinical or laboratory findings. In order to confirm the diagnosis radiologically two year-follow-up MRI was assessed. On the follow-up Modic type 1 in 8 of 27 patients partially converted Modic type 2 and 14 of 27 patients fully converted Modic type 2. Five of 27 patients were stable, and showed no change. CT slices were obtained from the diseased disc space and the affected vertebrae in all the patients to support the diagnosis.

Spondylodiscitis was diagnosed in the presence of paravertebral or epidural signal abnormalities with or without abscess formation. If such findings were absent, three of the following four criteria had to be fulfilled for the disk-space infection: signal abnormality of the bone marrow adjacent to the intervertebral disk (hypointense on T1-weighted images and hyperintense on T2-weighted images, signal not well demarcated); loss of the low-intensity vertebral endplate on T1-weighted images; hyperintensity of the disk on T2-weighted images; and disk enhancement after the injection of gadopentetate.

All patients gave a written informed consent to use their clinical data for the study purposes. The study protocol was approved at the research ethics review committee of the hospital.

### Imaging technique

A standard lumbar MRI protocol was employed to all patients. All images were acquired using on 1.5 Tesla MR units. All MR studies were performed on a 1.5 T unit (Magnetom Vision, Siemens, Erlangen, Germany) with gradient echo-planar capabilities and a standard phased array surface receiver coil for imaging the spine. The imaging protocol included axial and sagittal T1-weighted spin-echo sequences (552/12[TR/TE]), axial and sagittal T2-weighted turbo spin-echo (4000/120[TR/TE]) sequences, sagittal STIR(3600/60[TR/TE]) sequences, and sagittal diffusion-weighted sequences. Sagittal spinal DW images (b = 150 s/mm^2^) were acquired in the same plane and orientation as used in the routine sequences by using a reversed fast imaging with steady-state precession (PSIF) sequence (TR/TE 1.400 ms/100 ms; field of view 320×80 mm; section thickness 5 mm; intersection gap 0.5 mm; sections 6; matrix 128×256; echo train length 69 and one excitation) with spectral presaturation and inversion recovery (SPIR). In addition, axial and sagittal fat-suppressed T1-weighted images were obtained after IV infusion of 0.1 mmol/kg of gadopentate dimeglumine.

### Image assessment

All MR images were reviewed and evaluated by one radiologist specializing in MR imaging of the spinal system. The abnormal levels were classified as either infected or degenerative.

Signal intensity changes of the related disc and vertebral body marrow adjacent to the end plates of the degenerative spine on the conventional spin-echo sequence MR and the diffusion weighted MR were compared with those of spondylodiscitis.

We categorized the signal intensity of the abnormal vertebra on T1-Weighted images as hypointense relative to the presumed normal marrow. The signal of the abnormal vertebra on T2- weighted images was categorized as hypointense, isointense or hyperintense relative to the areas of the presumed normal marrow. On the diffusion-weighted images, the areas of the abnormal signal intensity were categorized as hypointense, isointense and hyperintense with respect to the normal marrow.

### Statistical analyses

The statistical analysis was carried out by using Statistical Package of Social Science (SPSS), version 13.0.

## Results

**FIGURE 1:**
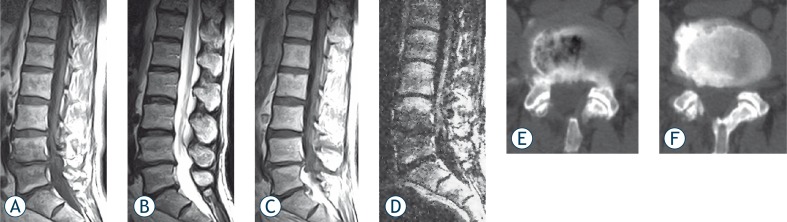
Sagittal MR images from 53 years old male patient with low back pain. A) T1-weighted MR image demonstrating low-signal intensity changes adjacent to the L4–5 disk, and B) T2-weighted MR image of the same level demonstrating high-signal intensity changes. C) On post-contrast T1-weighted images end-plates disclose signal intensity increase, which can also be seen in spondylodiscitis. D) On DWI end-plates showing low signal intensity changes consistent with Modic type 1 change at L4 through L5. E) and F) Axial CT slices obtained from the L4–5 disk level showing discal vacuum phenomenon and sclerosis, supporting the diagnosis of degenerative disc disease.

**FIGURE 2:**
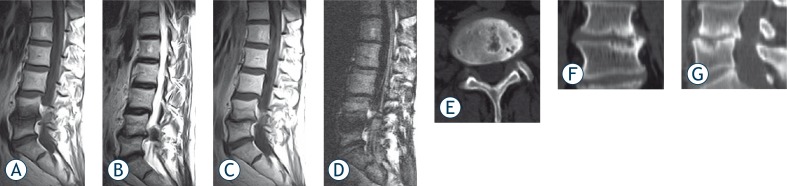
Sagittal MR images from 48 years old male patient with low back pain. A) Sagittal T1-weighted and B) Sagittal T2-weighted MR images showing low and high signal respectively at both end-plates of L4–5. C) On post-contrast T1-weighted images same level demonstrating heterogenic contrast enhancement. D) On DWI end-plates demonstrating low signal intensity changes consistent with Modic type 1 change at L4–5. E) Axial CT slices obtained from the L4–5 disk level and F) Coronal and G) Sagittal reconstructed CT image showing sclerosis on both end-plates of L4–5.

**FIGURE 3:**
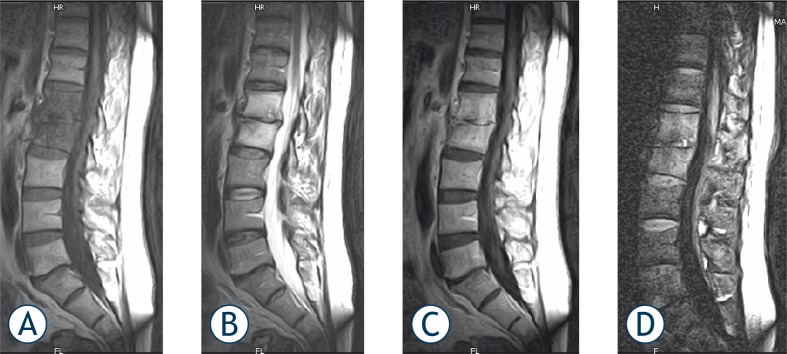
Sagittal MR images from 38 years old male patient with acute spondylodiscitis. A) T1-weighted image showing complete homogenous hypointensity at the L1 and L2 vertebrae corpus and adjacent intervertebral disc. There are also osteophytic changes on end-plate and loss of L1–2 intervertebral disc space. B) T2-weighted images showing hyperintense signal intensity corresponding to the same level. C) Postcontrast image showing homogenous enhancement of disc and adjacent vertebrae corpus. D) DWI revealing high signal intensity relative to neighbouring normal vertebrae.

The mean ages of patients with Modic type 1 change (Figures 1, 2) and spondylodiscitis (Figure 3) were 52.2 years (range, 24–77 years) and 55.8 years (range, 18–85 years), respectively.

These 27 patients had a total of 62 Modic changes and 18 patients with acute spondylodiscitis had 46 vertebral involvement. Four patients with type 1 Modic changes and 5 patients with spondylodiscitis had more than two vertebral involvements.

On CT slices of affected vertebrae in patients with Modic type 1 change, 5 patients (18.5%) had discal vacuum phenomenon, 22 patients (81%) had well-defined sclerosis, and 4 patients (14.8%) had erosions of vertebral endplates without bone destruction.

Bone marrow adjacent to the vertebral end plate in both Modic type 1 change and and acute spondylodiscitis were hypointense on T1-weighted images. The hypointense areas in the vertebral end plates on T1-weighted images in patients with Modic type 1 change enhanced either moderately or strongly on postcontrast images, a finding which could not be differentiated from a disc space infection. On T2-weighted images corresponding levels of vertebral end-plates showed hyperintense signal intensity in both groups. On diffusion-weighted MR images with relatively low b values, all vertebral body marrow and end-plates with Modic type 1 change showed hypointense to normal signal intensities. Conversely, all vertebral body marrow and end-plates with acute infectious spondylodiscitis showed increased signal intensities when compared to presumed normal vertebrae.

## Discussion

MR imaging (MRI) is commonly used in the diagnosis of patients with low back pain (LBP) and sciatica.^1^ In the search for causes of LBP, vertebral end-plate signal changes have come into focus. Vertebral end-plate changes are bone marrow and end-plate lesions visible in MRI.

Different pathological processes can involve vertebral bone marrow adjacent to the end-plates, including degenerative disc disease, infection and tumours, and these may present with a variety of signal intensity as shown by MRI.

After the initial description of Modic changes some studies have attempted to identify the cause of such changes. Modic type 1 changes were found to be associated with fissuring of the cartilaginous end-plate and increased vascularity within the subchondral bone marrow on the histological examination.^4^ Vertebral end-plate changes consistent with bone marrow oedema may also be seen in infective discitis, following intraosseous disc herniation (Schmorl’s nodules) and within 3 months of chemonucleolysis.^13^–^15^

Spondylodiscitis is an infection of the intervertebral disk and the adjacent vertebrae, with or without associated epidural or psoas abscesses. It is a serious disease both due to its long-term course and the possible outcomes.^16^

Type 1 Modic change and spondylodiscitis may both reveal similar symptoms, mainly LBP. Clinical and laboratory findings such as white blood cell count, erythrocyte sedimentation rate and elevated body temperature are, supportive but not confirmatory evidence in infectious spondylodiscitis.^17^

Stirling *et al*.^18^ suggested a theory that bacteria might play a causative role in LBP in Modic type 1 changes and that patients might benefit from the antibiotic treatment. Stirling *et al.*^18^ found bacteria in disc material from 19 of 36 patients with severe sciatica and Albert *et al*.^19^ showed that 17 out of 32 patients with Modic type 1 changes and persistent LBP achieved long-lasting pain relief after the long-term antibiotic treatment. But Wedderkopp *et al*.^20^ showed no evidence of bacteria in vertebrae in Modic type 1 changes in their recent study, although in this study possible presence of bacteria in the disc adjacent to the Modic type 1 changes in the vertebrae cannot be ruled out.

Fayad *et al*.^21^ found that patients with chronic LBP and predominantly type 1 Modic changes had a better short-term relief of symptoms following intradiskal steroid injection than those with predominantly type 2 changes, which further supports the inflammatory nature of Modic type 1 changes and the role of inflammation in the generation of LBP.

Although the aetiology of the inflammatory process in the vertebrae is still unknown in Modic type 1 change, there are two accepted possible theories.^20^ The most well known theory is that the process is a part of the “normal” degenerative process of the spine, where dehydration of the nucleus and loss of disc height biomechanically leads to unphysiological load and shear forces causing microfractures followed by inflammation in the vertebral end-plate and adjacent bone marrow.^20^ A second theory is that anaerobic bacteria with low virulence enter the vertebra from the bloodstream, resulting in infection, which is represented on MRI as Modic type 1 changes.^20^

On MRI, type 1 change reveals hypointensity on T1-weighted and hyperintensity on T2-weighted images. Affected bone marrow may show mild or strong contrast enhancement after the intravenous contrast administration. Differentiation of these findings from those of spondylodiscitis may not be always possible based on the conventional MRI findings alone.^6^,^21^,^22^ CT may be helpful in the differentiation showing discal vacuum phenomenon or well-defined sclerosis and erosions of vertebral endplates without bone destruction, findings that support the diagnosis of EIVO. In our patient group, CT revealed these changes compatible with EIVO at the end-plates adjacent to the degenerated disc.

DWI imaging has been successful to some extent in the differentiation of benign versus pathologic compression fractures of the vertebral body.^7^–^9^,^12^ It has been shown that benign bone marrow oedema has revealed hypointensity on DWI and malign vertebral compressions have disclosed hyperintensity in the vertebral bone marrow.^7^,^9^ Recently it has been reported that infective discitis has also shown hyperintensity on DWI as well as malignancies of the affected vertebral bone marrow.^12^ However, in this study we have found hypointensity on DWI at the Modic type 1 disorders.

In general, histopathologic findings of the type 1 bone marrow are fibrovascular tissue totally replacing normal marrow elements.^5^ A possible explanation for our results on diffusion-weighted MR imaging is that in the fibrovascular degenerative change, the increased free water of bone marrow caused by depletion of normal marrow elements leads to an increase in the extracellular volume fraction which produces low signal intensity in diffusion-weighted MR imaging. In contrast, in spondylodiscitis the reduction of the extracellular volume due to densly infiltrated inflammatory cells might lead to the increase in the signal intensity on diffusion-weighted MR imaging.

Although there are some clues for the diagnosis of type 1 changes on routine MRI, it sometimes may be very difficult to differentiate it from early onset spondylodiscitis. Intervertebral disk space infections typically give rise to vertebral marrow edema, manifesting as areas of low signal intensity on T1WI and high signal intensity on T2WI, thereby mimicking type 1 Modic changes. The contrast enhancement in the disk and endplates may occur in both conditions. Moreover, the enhancement of the intervertebral disc itself is not a definitive rule for spondylodiscitis, since sometimes this cannot be detected. Because of desiccation and dehydration, the disk often appears normal or hypointense on T2WI in degenerative disc disease, whereas its T2WI signal intensity is typically increased in spondylodiskitis.^21^ If there is enhancing paravertebral soft tissue mass adjacent to the intervertebral disc space, the MRI findings should orient the diagnosis toward an infectious process.^6^ The vertebral endplates are usually preserved in degenerative disc disease rather than destroyed or eroded with the bone destruction and the presence of these findings should also remind the possibility of disk space infection.^23^ However, in the absence of these findings, the decision should be made between EIVO type I and spondylodiscitis. We believe that DWI can make this differentiation easily and eliminate the necessity of CT in these groups of patients.

There are some limitations to the study presented here. The interpretations of the MR images were performed by only one experienced radiologist. Thus, the interobsever variability and accuracy associated with a less experienced radiologist was not assessed. Another limitation was the small number of patients who participated in the study. The diagnosis of Modic type 1 was relied on the clinical and radiological follow-up and we had no histopathologic correlation in our study. Further studies comparing histopathologic results and DWI findings are needed.

In clinical practice, most radiologic and clinical findings are sufficiently supportive for the differential diagnosis of pathological processes involving vertebral body marrow adjacent to the end-plates. But, sometimes Modic type 1 change causes a diagnostic dilemma in patients with low back pain since it has almost the same conventional MR imaging and clinical findings with the acute spondylodiscitis. Moreover postcontrast MR imaging will not lead to the differential diagnosis since the Modic type 1 change will also enhance as well as spondylodiscitis. However, DWI appears to be useful in the differentiation of EIVO from the acute spondylodiscitis with distinct documented features in this study.

For this reason, when results of clinical and conventional MR findings are equivocal, diffusion-weighted MR imaging may provide an excellent differential diagnosis between degenerative fibrovascular change of the spine and pyogenic spondylitis.
